# Genomic analysis of the international high-risk clonal lineage *Klebsiella pneumoniae* sequence type 395

**DOI:** 10.1186/s13073-023-01159-6

**Published:** 2023-02-13

**Authors:** Elvira R. Shaidullina, Michael Schwabe, Thomas Rohde, Valeria V. Shapovalova, Marina S. Dyachkova, Alina D. Matsvay, Yuliya A. Savochkina, Andrey A. Shelenkov, Yulia V. Mikhaylova, Katharina Sydow, François Lebreton, Evgeny A. Idelevich, Stefan E. Heiden, Karsten Becker, Roman S. Kozlov, German A. Shipulin, Vasiliy G. Akimkin, Michael Lalk, Sebastian Guenther, Andreas E. Zautner, Jürgen A. Bohnert, Ayslu M. Mardanova, Ruth Bouganim, Dror Marchaim, Katharina J. Hoff, Katharina Schaufler, Mikhail V. Edelstein

**Affiliations:** 1 Institute of Antimicrobial Chemotherapy, Smolensk State Medical University, Smolensk, Russia; 2 Pharmaceutical Microbiology, Institute of Pharmacy, University of Greifswald, Greifswald, Germany; 3 Federal State Budgetary Institution “Centre for Strategic Planning and Management of Biomedical Health Risks” of the Federal Medical Biological Agency, Moscow, Russia; 4 Central Research Institute of Epidemiology, Moscow, Russia; 5 Multidrug-Resistant Organism Repository and Surveillance Network, Walter Reed Army Institute of Research, Silver Spring, USA; 6 Friedrich Loeffler-Institute of Medical Microbiology, University Medicine Greifswald, Greifswald, Germany; 7 Institute of Medical Microbiology, University Hospital Münster, Münster, Germany; 8 Institute of Biochemistry, University of Greifswald, Greifswald, Germany; 9 Pharmaceutical Biology, Institute of Pharmacy, University of Greifswald, Greifswald, Germany; 10 Institute of Medical Microbiology and Hospital Hygiene, Medical Faculty, Otto-Von-Guericke University Magdeburg, Magdeburg, Germany; 11 Institute of Fundamental Medicine and Biology, Kazan Federal University, Kazan, Russia; 12 Department of Internal Medicine A, Shamir (Assaf Harofeh) Medical Center, Zerifin, Israel; 13 Infection Control Unit, Shamir (Assaf Harofeh) Medical Center and Sackler Faculty of Medicine, Tel Aviv University, Tel Aviv, Israel; 14 Institute of Mathematics and Computer Science, University of Greifswald, Greifswald, Germany; 15 Institute of Infection Medicine, Christian-Albrecht University Kiel and University Medical Center Schleswig-Holstein, Kiel, Germany

**Keywords:** *Klebsiella pneumoniae*, Recombination, Plasmid, Hypervirulence, Multidrug-resistance, Whole genome sequencing, Carbapenem resistance, ESBL

## Abstract

**Background:**

*Klebsiella pneumoniae*, which is frequently associated with hospital- and community-acquired infections, contains multidrug-resistant (MDR), hypervirulent (hv), non-MDR/non-hv as well as convergent representatives. It is known that mostly international high-risk clonal lineages including sequence types (ST) 11, 147, 258, and 307 drive their global spread. ST395, which was first reported in the context of a carbapenemase-associated outbreak in France in 2010, is a less well-characterized, yet emerging clonal lineage.

**Methods:**

We computationally analyzed a large collection of *K. pneumoniae* ST395 genomes (*n* = 297) both sequenced in this study and reported previously. By applying multiple bioinformatics tools, we investigated the core-genome phylogeny and evolution of ST395 as well as distribution of accessory genome elements associated with antibiotic resistance and virulence features.

**Results:**

Clustering of the core-SNP alignment revealed four major clades with eight smaller subclades. The subclades likely evolved through large chromosomal recombination, which involved different *K. pneumoniae* donors and affected, *inter alia*, capsule and lipopolysaccharide antigen biosynthesis regions. Most genomes contained acquired resistance genes to extended-spectrum cephalosporins, carbapenems, and other antibiotic classes carried by multiple plasmid types, and many were positive for hypervirulence markers, including the siderophore aerobactin. The detection of “hybrid” resistance and virulence plasmids suggests the occurrence of the convergent ST395 pathotype.

**Conclusions:**

To the best of our knowledge, this is the first study that investigated a large international collection of *K. pneumoniae* ST395 genomes and elucidated phylogenetics and detailed genomic characteristics of this emerging high-risk clonal lineage.

**Supplementary Information:**

The online version contains supplementary material available at 10.1186/s13073-023-01159-6.

## Background

*Klebsiella pneumoniae* is an opportunistic pathogen and one of the most common causes of hospital- and community-acquired infections [1]. It has been traditionally differentiated into classic (frequently but not exclusively multidrug-resistant [MDR]) and hypervirulent *K. pneumoniae* (hvKp) with the latter appearing clinically as mostly antibiotic-susceptible and invasive pathogen able to induce multiple site infection [2–4]. However, a recent study revealed that definition boundaries are blurred and that a strict assignation to particular contexts (hospital- or community-associated) not always applies [5]. Various virulence-associated features enable some *Klebsiella* strains’ pathogenicity including significant siderophore secretion and hypermucoviscosity [6]. The presence of certain biomarkers, for example, *peg-344* (metabolite transporter), *iucA* (aerobactin), *iroB* (salmochelin), and *rmpA* (regulator hypermucoviscosity), has been shown to predict hypervirulence phenotypes and distinguish hvKp from non-hvKp strains [6, 7]. However, the abovementioned study [5] also suggests that for some settings, these markers are rather unreliable.

Several sequence types (ST) and clonal lineages have been associated with either antimicrobial resistance (AMR) or hypervirulence characteristics; however, the convergence of both has also been increasingly reported during the last few years [8]. The emergence of isolates resistant to most or even all clinically available antibiotics yet exacerbates the overall tense situation and is mostly driven by the acquisition of mobile resistance genes [9]. This includes, for example, the production of the plasmid-encoded extended-spectrum beta-lactamase (ESBL) and carbapenemase (KPC, NDM, and OXA-48) enzymes as well as mobilizable colistin resistance (*mcr*) genes [10]. MDR *K. pneumoniae* isolates often belong to clonal complex (CC)258 (including ST11, ST258, ST340, and ST512) and other established high-risk clonal lineages: ST15, ST101, ST147, and ST307, among others, as defined by multilocus sequence typing (MLST) [11–14], that disseminate globally and are often involved in nosocomial outbreaks [15]. A so far less well-characterized genetic lineage, ST395, is an emerging international high-risk clonal lineage that has been associated with MDR and found to harbor ESBL and carbapenemases, as well as many other resistance determinants including 16S rRNA methyltransferases conferring pan-aminoglycoside resistance [16]. ST395 differs from ST11, the closest member within CC258, in three of seven MLST loci and, therefore, does not belong to CC258 by classical MLST definition. However, genome-wide analysis of ST395 revealed that it has likely evolved from ST11 through recombination and acquisition of large genomic regions from unrelated STs, and thus, in a broader sense, it may be considered a sub-lineage within CC258 [17]. ST395 was first reported from an outbreak of OXA-48 producing *K. pneumoniae* in a French hospital in 2010 [18]. Shortly after, in 2011, OXA-48 producing isolates from The Netherlands and Morocco were linked to the same sequence type/clone based on MLST and repetitive element sequence-based PCR [19]. ST395 isolates carrying OXA-48 plasmids have spread widely and were recovered either sporadically or from outbreaks in many countries in Europe (the Czech Republic, Denmark, France, Germany, Hungary, Ireland, Italy, Romania, Russia, Sweden, the UK), North Africa (Egypt, Algeria), Southeast Asia (Malaysia), and the Middle East (Israel, Kuwait) [18–37]. Besides OXA-48, isolates of this genotype have been reported to carry different carbapenemases, such as KPC-2 in China [38], KPC-3 in Italy [39], NDM-1 in Russia [40, 41], and NDM-1 and NDM-5 in Germany [8]. Most of the above reports noted simultaneous production of carbapenemases and ESBL, predominantly of the CTX-M group.

This study aimed to genomically characterize the *K. pneumoniae* ST395 clonal lineage in-depth by using different bioinformatics approaches including phylogenetics, genome evolution, and distribution of acquired AMR and virulence genes.

## Methods

### Data origin

A sample library of 297 *K. pneumoniae* ST395 genomes (assembled genomes and paired-end reads) was acquired, including both previously unpublished and publicly available data. A list of all genomic data used, and their origin, can be found in Table 1 and Additional file 1: Table S1. Whole genome sequence data were either provided by collaborating partners (*n* = 138) or downloaded from public databases (*n* = 159). For the former, a total of 121 new whole genome sequences of ST395 isolates with the corresponding metadata were obtained, which included 108 isolates collected from 20 cities across Russia between 2013 and 2018, ten isolates collected from three cities of Belarus in 2014, and three isolates collected from one city in Kazakhstan in 2017. All these isolates were referred to the central laboratory of the Institute of Antimicrobial Chemotherapy (IAC) as part of the national sentinel AMR surveillance program [42]. In this surveillance, each participating center (hospital) contributed annually up to 150 consecutive non-duplicate (one per patient/case of infection) clinical isolates of different species recovered from representative specimens (blood, cerebrospinal fluid, tissue biopsies, bronchoalveolar lavage, endotracheal aspirate, sputum, urine, etc.) of hospitalized patients with clinical symptoms of infection. Isolates recovered from patients without clinical symptoms of infection or from hospital environment for epidemiological screening purposes, as well as repeated isolates of the same species recovered from the same patient, were spared. In the IAC laboratory, the species identity of isolates was confirmed by MALDI-TOF mass spectrometry with the Microflex LT-MALDI Biotyper System (Bruker Daltonics, Bremen, Germany), susceptibility to various antibiotics, including meropenem, was determined by broth microdilution method according to ISO 20776–2:2021 [43] and EUCAST [44] methodology, and the presence of carbapenemase genes was assessed by means of real-time PCR in all *Enterobacterales* isolates with decreased susceptibility to meropenem (MIC > 0.125 mg/L) as recommended by EUCAST [45]. A maximum of 20 carbapenemase gene-positive *K. pneumoniae* isolates consecutively collected from each hospital participating in the surveillance were then selected for genome sequencing. Short-read paired-end sequencing of these isolates was performed on Illumina platforms and then used to construct draft genome assemblies. For a subset of isolates, long-read sequencing on MinION (Oxford Nanopore Technologies) was additionally performed to obtain hybrid genome assemblies. The genome sequences of ST395 isolates from Russia, Kazakhstan, and Belarus obtained for this study were submitted to the European Nucleotide Archive under the project accession number PRJEB49683 [46].

**Table 1:** Summary of the whole genome sequences used in this study

Country	Genome source^a^	Isolation source^b^	Carbapenemase gene-positive	ESBL and/or AmpC gene-positive	16S rRNA methyltransferase gene-positive	Hypervirulence gene-positive
Bangladesh	- / 1	1 / - / - / -	1	1	1	-
Belarus	10 / 10	20 / - / - / -	15	18	15	6
Brazil	- / 1	1 / - / - / -	1	-	-	-
China	- / 8	4 / 3 / - / 1	5	8	6	3
Estonia	- / 10	10 / - / - / -	-	10	-	-
Finland	- / 1	1 / - / - / -	1	1	-	1
France	- / 1	1 / - / - / -	-	-	-	-
Georgia	- / 1	1 / - / - / -	1	1	-	-
Germany	1 / 8	9 / - / - / -	6	8	2	3
India	- / 7	7 / - / - / -	5	3	3	1
Israel	- / 7	7 / - / - / -	1	7	1	-
Italy	- / 13	13 / - / - / -	10	10	-	10
Kazakhstan	3 / -	3 / - / - / -	3	3	3	3
Lebanon	- / 1	- / - / 1 / -	-	-	-	-
Lithuania	- / 1	1 / - / - / -	-	1	1	-
Luxembourg	- / 2	1 / - / - / 1	-	2	-	-
Netherlands	- / 1	- / - / - / 1	-	1	-	-
Nigeria	- / 7	7 / - / - / -	6	7	-	-
Portugal	- / 1	1 / - / - / -	-	1	-	-
Romania	- / 5	1 / - / 4 / -	4	5	-	-
Russia	108 / 59	163 / - / - / 4	153	147	66	102
South Korea	- / 4	4 / - / - / -	-	4	-	-
Switzerland	- / 2	2 / - / - / -	2	2	2	-
Turkey	- / 8	8 / - / - / -	8	6	8	-
Ukraine	- / 3	3 / - / - / -	3	3	-	-
USA	- / 9	9 / - / - / -	2	8	1	1
Vietnam	- / 4	4 / - / - / -	3	4	4	-
Total:	122 / 175	282 / 3 / 5 / 7	230	261	113	130

^a^Genome Source: This study / Public databases

^b^Isolations source: Human / Animal / Environment / Not available

In addition, collaboration partners provided previously unpublished sequence read data of 17 isolates from Israel, the United States (US) of America and Germany. The sequence read data from Israel comprised six isolates (020-1_S1, 020-12_S12, 020-3_S1, 020-4_S4, 020-7_S7, 020-8_S8), which have been previously described by Bouganim *et al.* [47]. The nine genomes from the USA corresponded to isolates from six American patients (MRSN31307, MRSN31314, MRSN31330, MRSN31332, MRSN31333, MRSN8909), one German patient (MRSN363431), and two Ukrainian patients (MRSN567231, MRSN740795). The two German isolates were MRGN_20, which has been previously described by Zautner *et al.* [48] and PBIO2028, a clinical isolate from Greifswald. The associated metadata is listed in Additional file 1: Table S1. Raw reads for the isolates from Israel and Germany were submitted to the European Nucleotide Archive under the project accession number PRJEB53512 [49]. Assemblies of the US samples have been deposited with links to BioProject accession number PRJNA849970 [50] in the NCBI BioProject database (https://www.ncbi.nlm.nih.gov/bioproject/).

Additionally, to obtain already published *K. pneumoniae* ST395 genomic data, first, literature research was performed using the keywords: “*Klebsiella pneumoniae*” or “*K. pneumoniae*” and “sequence type 395” or “ST395.” Then, paired-end reads were downloaded, upon availability. Second, the NCBI (National Center for Biotechnology Information) assembly database was searched with the taxonomic ID of *Klebsiella pneumoniae* “taxid573.” The sequence types (ST) of genomes were determined using MLST (v. 2.19.0) [51]. For ST395 genomes, the NCBI’s Short Read Archive (SRA) was searched for paired-end read data. If no paired-end reads were available, the available assemblies were used for further analysis.

### Short-read whole genome sequencing

For the abovementioned whole genome-sequenced isolates, extracted genomic DNA was used for library preparation after preliminary ultrasonic fragmentation using M220 Focused-ultrasonicator (Covaris, USA). Repair and dA-tailing of DNA fragments was performed using the NEBNext Ultra II End Repair / dA-Tailing Module (New England Biolabs, USA) according to the manufacturer’s protocol. Ligation of 0.25 µM KAPA DI Adapters (Roche Diagnostics GmbH, Germany) was performed overnight using T4 DNA ligase (New England Biolabs, USA). Libraries were amplified using the DreamTaq PCR Master Mix (2x) (Thermo Fisher Scientific, USA) according to the manufacturer’s protocol, PCR was stopped at saturation. All clean-up and size selection procedures were carried out using AMPure XP beads (Beckman Coulter Life Sciences, USA) according to the manufacturer’s protocol. Quality of libraries was assessed by capillary electrophoresis using the Agilent 2100 Bioanalyzer system (Agilent, USA). Library concentration was measured as described above. Sequencing was performed on the Illumina NextSeq 550 System with the NextSeq 500/550 Mid Output Kit v2.5 (300 Cycles) (Illumina, USA).

### Long-read whole genome sequencing

Extracted genomic DNA was used for library preparation with NEBNext Companion Module (New England Biolabs, USA) and Ligation Sequencing Kit (Oxford Nanopore Technologies, UK) in accordance with the manufacturer’s protocol. Samples were multiplexed using Native Barcoding Expansion 1–12 (Oxford Nanopore Technologies, UK). Library concentration was measured with the Qubit 4 Fluorometer (Thermo Fisher Scientific, USA) using the Qubit dsDNA HS Assay Kit (Thermo Fisher Scientific, USA). Sequencing was performed on the MinION platform (Oxford Nanopore Technologies, UK) using the R9.4.1 Flow Cell.

### Data quality and processing of raw data

Raw sequencing reads were quality checked with FastQC [52]. Adapter sequences and low-quality bases were trimmed with Trimmomatic v.0.39 run in paired-end mode [53]. After trimming, eukaryotic contaminations were identified with KRAKEN2 (v.2.1.2) [54] using the standard database (v.2021.05.17). The KRAKENTOOLS toolkit (v.1.2) was used to remove the eukaryotic reads (taxid: 2759) from the paired-end FASTQ files. De novo genome assemblies of contamination-filtered reads were performed with SPAdes [55]. The resulting contig files were used for further analyses.

Base-calling and demultiplexing of Oxford Nanopore data were conducted using Guppy v.5.0.16 with the “sup” model. The quality control was performed with MinIONQC (v.1.4.2) [56]. Hybrid assemblies were prepared by using the Trycycler pipeline v.0.5.1 [57]. The consensus contigs were polished with Medaka v.1.4.4 [58]. Short-read polishing was performed by Polypolish tool [59] and POLCA script [60].

### Processing of assembled data

The contamination removal method was based on determining coverage cutoffs for each assembly after visual inspection of coverage-versus-length (CVL) plots as described by Douglass *et al.* [61]. The k-mer coverage value was provided by SPAdes assembler.

### Phylogenetic analyses

We performed core-genome SNP analysis of all the genomes against the previously published, fully assembled genome of PBIO1951 using Parsnp v.1.5.6 [62]. PBIO1951 has been selected due to its complete genome and the described epidemiologic link between St. Petersburg (Russia) and Greifswald (Germany) [8]. The core-genome alignment was clustered using BAPS with the fastBAPS R package 1.0.6 [63]. Visualization and annotation of phylogenetic trees were performed using iTOL v.6 [64].

### Identification of large recombination events and putative donors

The complete genome of the PBIO1951 isolate was used as the reference genome. All other ST395 genomes from the dataset were mapped to the reference genome using Snippy [65]. Putative regions of recombination were predicted by Gubbins [66]. The Gubbins output files were used to calculate the mean recombination count per base (Additional file 2).

The method described by Comandatore *et al.* in [67] was then used to identify the donors of the large recombination events detected within ST395. Briefly, a total of 1303 complete genome assemblies of *K. pneumoniae* isolates representing different STs were retrieved from the Patric database [68]. They were merged to the ST395 dataset and aligned to the reference genome of PBIO1951. The core SNPs were called again using Snippy, and those corresponding to each recombination region of > 100 kbp predicted by Gubbins were extracted and subjected to separate phylogenetic analysis using the FastTree tool [69, 70] with 100 resamples.

Each resulting tree was manually analyzed as follows: (i) the ST395 recipients of the recombination were identified on the tree and (ii) when a monophylum including all the recipients and one or more non-ST395 strains was detected, the latter were considered as putative donors of the recombination.

### Identification of virulence, antimicrobial resistance genes, and essential genetic elements

Identification of open reading frames (ORFs) and gene contents in the assembled genomes was performed using Prokka v.1.14.6 [71]. Antimicrobial resistance genes were annotated using AMRFinderPlus tool v.3.10.5 [72] with BLASTP, BLASTX, and HMMER algorithms. Detection of virulence factors was performed by using the ABRicate program v.1.0.1 with BIGSdb-Kp virulence typing databases [73, 74]. Kleborate v.2.1.0 [75] and Kaptive [76] databases were used for MLST, capsule polysaccharide (K), and lipopolysaccharide (O) antigen molecular typing. The K-locus was classified as “ambigious” when Kaptive was unable to confidently identify a K-locus due to fragmented or incomplete genome assemblies, or the presence of atypical *wzi* allelic variants or of atypical gene composition of a K-locus. BIGSdb-Kp [77] online tools and databases were used for core-genome MLST (cgMLST) and virulence typing (YbST) and for identification of ICE*Kp* elements [12, 74]. Insertion sequences were determined by ISfinder online tool [78]. Prophages were searched using the online PHASTER tool [79]. Partially matching sequences, point mutations, and indels were further manually curated by using QIAGEN CLC Genomics Workbench 22.

### Plasmid analysis

MOB-suite with MOB-recon and MOB-typer software modules [80] was used to partially reconstruct and type the plasmids carrying the carbapenemase and aerobactin cluster genes from short-read genome assemblies. Representative plasmid sequences shown in Fig. 4 were extracted from hybrid short- and long-read assemblies obtained using Trycycler as described above and visualized with the BLAST Ring Image Generator (BRIG) tool v.0.95 [81] and NCBI BLAST + v.2.12.0 [82].

A detailed overview of the used programs and settings can be found in Additional file 3: Detailed Methods.

## Results

### ST395 genomes were obtained from different countries with the majority originating from Russia

Overall, we included 297 *K. pneumoniae* ST395 genomes from 27 different countries and four continents, which were either collected and sequenced in this study or had sequence data available in public repositories. Most of the Russian isolates (108/167; 64.7%) were collected as part of the national sentinel AMR surveillance program [42] and subjected to whole genome sequencing to identify carbapenemase-producing *K. pneumoniae*. Therefore, the sample set was also biased towards carbapenemase- and MDR-positive isolates. Human clinical isolates represented the vast majority (282/297; 94.9%) compared to environmental (5/297; 1.7%) and animal (3/297; 1%) isolates (for the remaining 7/297 [2,4%] genomes retrieved from public databases, source information was missing [Table 1]).

The documented diseases from which the human clinical isolates were recovered included respiratory (52/199; 26.1%), urinary (46/199; 23.1%), bloodstream (42/199; 21.1%), skin and soft tissue (14/199; 7%), and other (14/199; 7%) infections (Additional file 1: Table S1).

### ST395 subclades evolved through recombination and insertion of large chromosomal regions

**Fig. 1:**
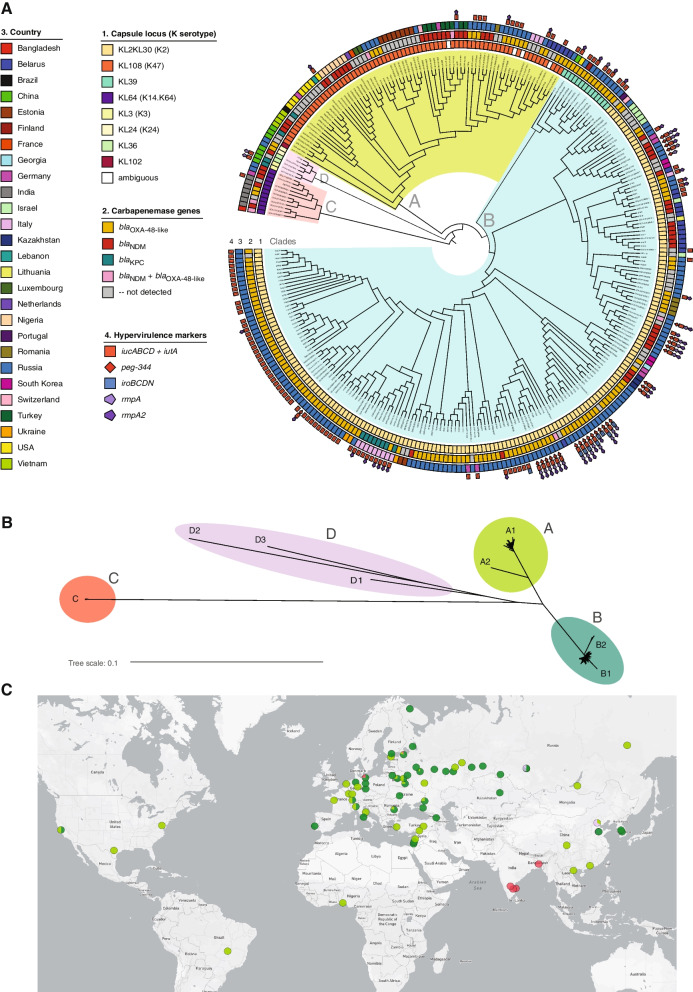
Core-genome SNP phylogeny and geographic distribution of ST395. Phylogenetic tree of international ST395 isolates inferred from core-genome SNP analysis by Parsnp and visualized with iTOL. **A** Midpoint-rooted tree with unscaled branch lengths shows the classification of isolates into four clades and their characteristics according to the color legend. **B** Unrooted tree with scaled branch lengths shows the evolutionary distance between clades and subclades. **C** The map shows the geographic distribution of clades

Alignment of all ST395 genomes against the fully assembled PBIO1951 reference revealed a common core genome of about 3.87 Mb with a total of 12,379 SNP positions identified by Parsnp analysis that were used to construct the phylogenetic tree (Fig. 1). BAPS clustering of the core-SNP alignment assigned all genomes to four well-separated clades designated A, B, C, and D, and eight smaller subclades named A1, A2, B1, B2, C, D1, D2, and D3, respectively. The minimum inter-clade distances (expressed as the number of SNPs between the closest representatives of two clades) ranged from 765 for clades A and B (median distance: 810.0) to 3034 for clades C and D (median distance: 3499.0) (Fig. 1B).

**Fig. 2:**
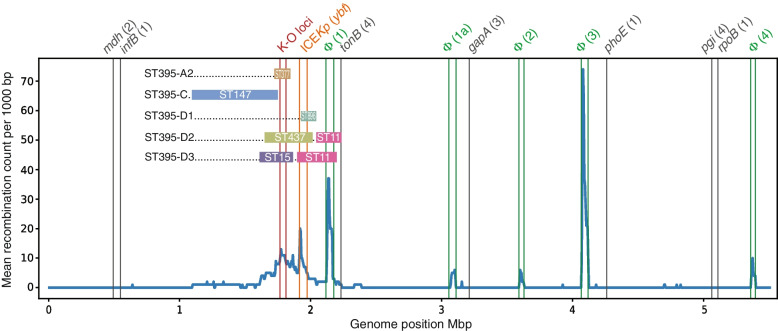
Mean recombination counts across ST395 chromosomes and putative large recombination events. The positions of seven MLST loci are indicated by gray vertical lines with corresponding allele names and numbers in brackets shown on top. K and O loci boundaries are indicated by red vertical lines. The ICE*Kp* element and prophage integration regions are bounded, respectively, by orange and green vertical lines. Prophages are denoted by the “Φ” symbol followed by a number in brackets: 1 and 1a, similar to *Salmonella* phage SEN34 (NC_028699); 2, similar to *Klebsiella* phage ST512-KPC3phi13.2 (NC_049452); 3, similar to *Edwardsiella* phage GF-2 (NC_026611); 4, similar to *Klebsiella* phage 3LV2017 (NC_047817). Large subclade-specific recombination regions are shown by colored horizontal bars indicating the putative donor lineages

We observed a significant correlation between phylogenetic clustering and the landscape of certain chromosomal markers (Fig. 2). Most notably, we found strict concordance between the type of capsule synthesis locus (KL), which determines the capsule serotype (K-type), and the subclades within ST395. Thus, all but four isolates of subclade A1 carried the KL108 (K47 type) (the remaining four isolates showed recombinations in the K-locus and, therefore, were not assigned to a particular K-type). The only isolate of subclade A2 carried KL102. All isolates of subclade B1 carried KL2KL30 (K2 type), while the isolates of subclade B2 showed the KL39 type. While the clade C-genomes showed the KL64 (K14.K64 type) type, the D1-, D2-, and D3-subclade isolates demonstrated the KL3 (K3 type), KL36, and KL24 (K24 type) types, respectively (Fig. 1A).

Based on these results, we explored the hypothesis that, similar to other *K. pneumoniae* MDR clonal lineages [17, 83], ST395 diverged into distinct clades and subclades through chromosomal recombination, which eventually led to the observed capsule locus diversity. Analysis of the entire genome collection by Gubbins revealed a total of 678 putative recombination events, twelve (1.8%) of which involved large (> 100 kbp) regions. Most recombination events occurred in chromosomal regions but did not affect any of the MLST loci; however, the largest recombination events spanned a wide (1.1 Mbp) area of the chromosome which included capsule (K) and LPS antigen (O) biosynthesis loci (Fig. 2). In this area, the core-SNP profiles as well as core-genome MLST (cgMLST) profiles showed considerable diversity among different subclades (Additional file 1: Table S2). We therefore used two different approaches to identify putative recombination donors: (i) a core-SNP-based phylogenetic approach, which involved analysis of *K. pneumoniae* collections of genomes retrieved from the Patric database, and (ii) a query by cgMLST locus combination for the putative recombination regions in the BIGSdb database. While we did not identify donors for all ST395 clades, we found that the ST147 *Klebsiella* lineage was involved in one or more recombination events, which resulted in the acquisition of a large fragment (> 661 kbp) by ST395 clade C within the genomic area mentioned above. We also identified fragments of 101 to 388 kbp spanning the same area that were likely acquired from donors of other *Klebsiella* sequence types: ST377 in subclade A2, ST896 in subclade D1, ST437 and ST11 in subclade D2, and ST15 and ST11 in subclade D3 (Fig. 2, Additional file 4). As expected, the regions corresponding to K and O loci (highlighted in red vertical lines in Fig. 2) showed peaks in recombination counts. Another major peak defining a recombination hot-spot within this area corresponded to the integrative conjugative element ICE*Kp* (highlighted in orange vertical lines in Fig. 2), which encodes the siderophore yersiniabactin and its receptor (*ybt* locus). We detected different ICE*Kp* elements that represent distinct *ybt* lineages in 223 of 297 (75.1%) ST395 isolates. ICE*Kp12* (*ybt16*) was the predominant type, detected in 182 of 189 (96.3%) genomes of subclade B1 as well as all 14 isolates of subclade B2 and eleven isolates of clade C. Other ICE*Kp* variants were less common: ICE*Kp11* (*ybt15*) was found in three of 76 (3.9%) isolates of subclade A1 and both genomes of subclade D1; ICE*Kp5* (*ybt14*) in six of 76 (7.9%) isolates of subclade A1; ICE*Kp2* (*ybt13*) in both isolates of subclade D3; ICE*Kp4* (*ybt10*), ICE*Kp3* (*ybt9*), and the unclassified ICE*Kp* variant in one isolate each of subclades A1, B1, and B1, respectively. Sixty-six of 76 (86.8%) isolates of subclade A1, the only isolate of subclade A2, 5/189 (2.6%) isolates of subclade B1, and both isolates of subclade D2 lacked ICE*Kp*. Finally, we identified eight regions in the chromosome that showed peaks in recombination frequency associated with prophage-related sequences. Five of these regions (highlighted in green vertical lines in Fig. 2) were called by PHASTER as a questionable complete phage (Φ1: similar to *Salmonella* phage SEN34 [NC_028699]) or intact phages (phages Φ1a, Φ2, Φ3, and Φ4: similar, respectively, to SEN34, *Klebsiella* phage ST512-KPC3phi13.2 [NC_049452], *Edwardsiella* phage GF-2 [NC_026611], and *Klebsiella* phage 3LV2017 [NC_047817]).

### ST395 isolates often carried carbapenemase and ESBL genes simultaneously

**Fig. 3:**
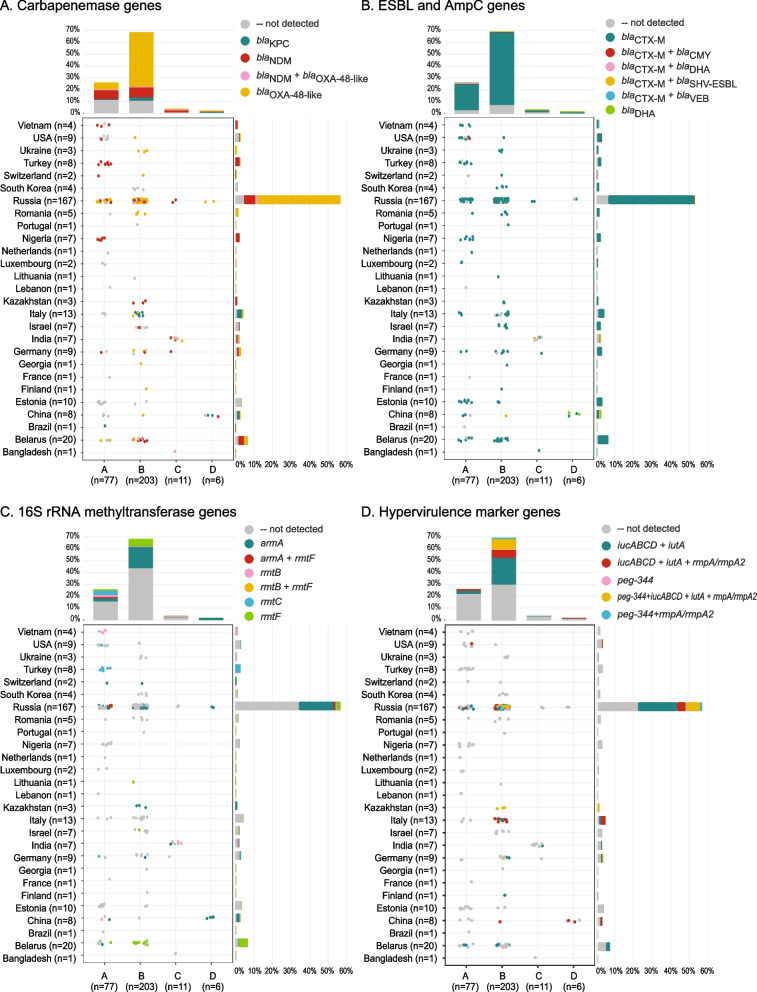
Distribution of major antibiotic resistance and hypervirulence markers by ST395 clades and countries. Data shown summarizes distribution of carbapenemase genes (**A**), ESBL and AmpC genes (**B**), 16S rRNA methyltransferase genes (**C**), and hypervirulence marker genes (**D**) by country and clade. Each circle represents a genome colored based on the marker gene (combination). Barplots summarize the percentage of genomes from each clade (top) and country (right) and are colored based on the marker gene (combination)

Characterization of acquired resistance genes, especially carbapenemase, extended-spectrum β-lactamase (ESBL), and molecular class C cephalosporinase (AmpC) genes, was one of the other interests of this study (Table 1, Fig. 3A, B). The genes of the OXA-48 family carbapenemases (*bla*_OXA-48-like_) were the most common, found in 163 of 297 (54.9%) of all isolates. The dominant variant, *bla*_OXA-48_, was identified in the majority (133/167; 79.6%) of the Russian isolates but also in isolates from other European countries. The less common variants, *bla*_OXA-232_ and *bla*_OXA-244_, encoding enzymes with weaker carbapenem-hydrolyzing activity were identified, respectively, in the genomes of four isolates from India and Bangladesh, and two isolates from Russia. The genes of the NDM family carbapenemases (*bla*_NDM_) were the second most prevalent (61/297; 20.5%) in isolates of all ST395 clades from various world regions. These included the most typical *bla*_NDM-1_ gene (52/297; 17.5%) detected in Belarus, Germany, Israel, Kazakhstan, Nigeria, Russia, Switzerland, Turkey, USA, and Vietnam, *bla*_NDM-5_ (8/297; 2.7%) in Bangladesh, China, Germany, India, and Russia, and *bla*_NDM-16_ in one (0.3%) Russian isolate. The genes for KPC type carbapenemases (*bla*_KPC_) were the least common: *bla*_KPC-2_ (4/297; 1.3%) was found in the isolates of different ST395 clusters from Brazil and China, and *bla*_KPC-3_ (8/297, 2.7%) in closely related isolates from Italy. Six isolates (2%) from Bangladesh, India, Russia, and Turkey were found to carry a combination of *bla*_NDM-1_ and one of the *bla*_OXA-48-like_ genes. Carbapenemase-negative ST395 isolates occurred in many countries (Fig. 3A).

ESBL genes were almost universally present among global ST395 isolates (Fig. 3B). The majority (259/297; 87.2%) possessed the CTX-M-gene family, of which *bla*_CTX-M-15_ was the most common type (247/297; 83.2%). Other ESBL genes were detected sporadically: *bla*_CTX-M-27_ (8/297; 2.7%), *bla*_CTX-M-3_ (4/297; 1.3%), *bla*_SHV-31_ (2/297; 0.7%), *bla*_SHV-2_, *bla*_VEB-1,_ and *bla*_CTX-M-55_ (1/297; 0.3% each). In total 68.0% (202/297) of the isolates carried carbapenemase and ESBL resistance genes simultaneously. Six (2%) isolates representing different countries and ST395 clades carried the genes for acquired AmpC cephalosporinases: *bla*_DHA-1_ (4/297; 1.3%), *bla*_CMY-2_ and *bla*_CMY-6_ (1/297; 0.3% each), either alone or in combination with *bla*_CTX-M_ (Fig. 3B).

### ST395 carried an unusually high abundance and diversity of 16S rRNA methyltransferase genes

Besides β-lactamases, we identified a great variety of resistance genes of plasmid and/or chromosomal origin to all antibiotic classes in the ST395 genomes (Table 1, Additional file 1: Table S1). Some of these genes, such as the dual-targeting aminoglycoside- and fluoroquinolone-modifying acetyltransferase genes (*aac(6')-Ib-cr*), were highly common (232/297; 78.1%), while others, such as the mobile colistin resistance gene (*mcr-8.2*), were found in single isolates only. Besides *aac(6')-Ib-cr*, ST395 isolates carried many other quinolone resistance determinants, including genes of the quinolone family protective proteins (*qnrB*, *qnrD1*, and *qnrS1*), resistance-nodulation-cell division (RND) and major facilitator superfamily (MFS) efflux pumps (*oqxA*, *oqxB*, and *qepA*). All, except for ten isolates, also harbored a variety of genes of aminoglycoside-modifying enzymes in various combinations. Most notably, however, we found an unusually high abundance (113/297; 38.1%) and diversity of 16S rRNA methyltransferase genes, which confer resistance to all aminoglycosides (Fig. 3C). ArmA was the most prevalent (71/297; 23.9%) type of methyltransferase detected. Isolates carrying *armA* genes represented all ST395 subclades and seven countries of origin. Other types of methyltransferase genes, in order of decreasing prevalence, *rmtF* (24/297; 8.1%), *rmtC* (12/297; 4%), and *rmtB* (10/297; 3.4%), were more specifically associated with certain countries and geographic regions. For instance, *rmtF* and *rmtC* were most common among the isolates from Belarus and Turkey, respectively, but occurred only sporadically in the isolates from other countries. The genomes of four (1.3%) Russian isolates revealed the simultaneous presence of two methyltransferase genes (*rmtF* and either of *armA* or *rmtB*).

### Hypervirulence markers were mostly found in ST395 clade B genomes

Next, we explored the distribution of hypervirulence (hv) genes in ST395. While the studied genomes contained many different biomarkers that may contribute to virulence in *K. pneumoniae* (Table 1, Additional file 1: Table S1), we assessed more specifically the plasmid virulence markers that have been identified as most important for differentiating the hvKp from non-hvKp representatives, namely, the aerobactin (*iucABCD-iutA*) and salmochelin (*iroBCDN*) iron uptake systems, regulators of mucoid phenotypes (*rmpA* and *rmpA2*), and the metabolite transporter gene (*peg-344*) [6]. These biomarkers were detected in 130 of 297 (43.8%) of the isolates but were not in complete linkage disequilibrium with each other (Fig. 3D). Aerobactin synthesis and receptor genes were detected in 126 of 297 (42.4%) isolates, either alone (74/297; 24.9%) or in combination with other markers. *rmpA* and *rmpA2* in 51 of 297 (17.2%), *peg-*344 in 26/297 (8.8%), and the salmochelin gene cluster in six of 297 (2%) only. The distribution of these biomarkers among different ST395 clades and countries was rather uneven. Most isolates carrying hv genes belonged to clade B (115/130, 88.5%) and originated from Russia (102/130; 78.5%), Italy (10/130; 7.7%), and Belarus (6/130; 4.6%) (Fig. 3D).

### Carbapenemase genes were associated with different plasmid replicon types and occasionally located on “hybrid” plasmids

Using MOB-suite, we were able to partially reconstruct and characterize the plasmids carrying carbapenemase genes for 202 draft genome assemblies of overall 230 carbapenemase-positive isolates (87.8%) (Additional file 1: Table S3). Carbapenemase genes were tentatively associated with plasmids of 14 different replicon types with the highest diversity observed for *bla*_NDM_-carrying plasmids (nine types) and lower diversity for *bla*_OXA-48-like_- and *bla*_KPC_-harboring plasmids—five and three types, respectively. *bla*_OXA-48-like_ genes were found predominantly on IncL (80/145; 55.2%) and IncM2 plasmids (56/145; 38.6%), *bla*_NDM_ genes were mostly located on multi-replicon plasmids of IncFIB/IncFII (22/53; 41.5%) and IncFIB/IncHI1B types (16/53; 30.2%), and *bla*_KPC-3_ genes were always associated with pKPC_Kp02-like plasmids. While we did not attempt to systematically assemble the plasmids carrying ESBL genes, we found different replicon types associated with ESBL and AmpCs (data not shown).

**Fig. 4:**
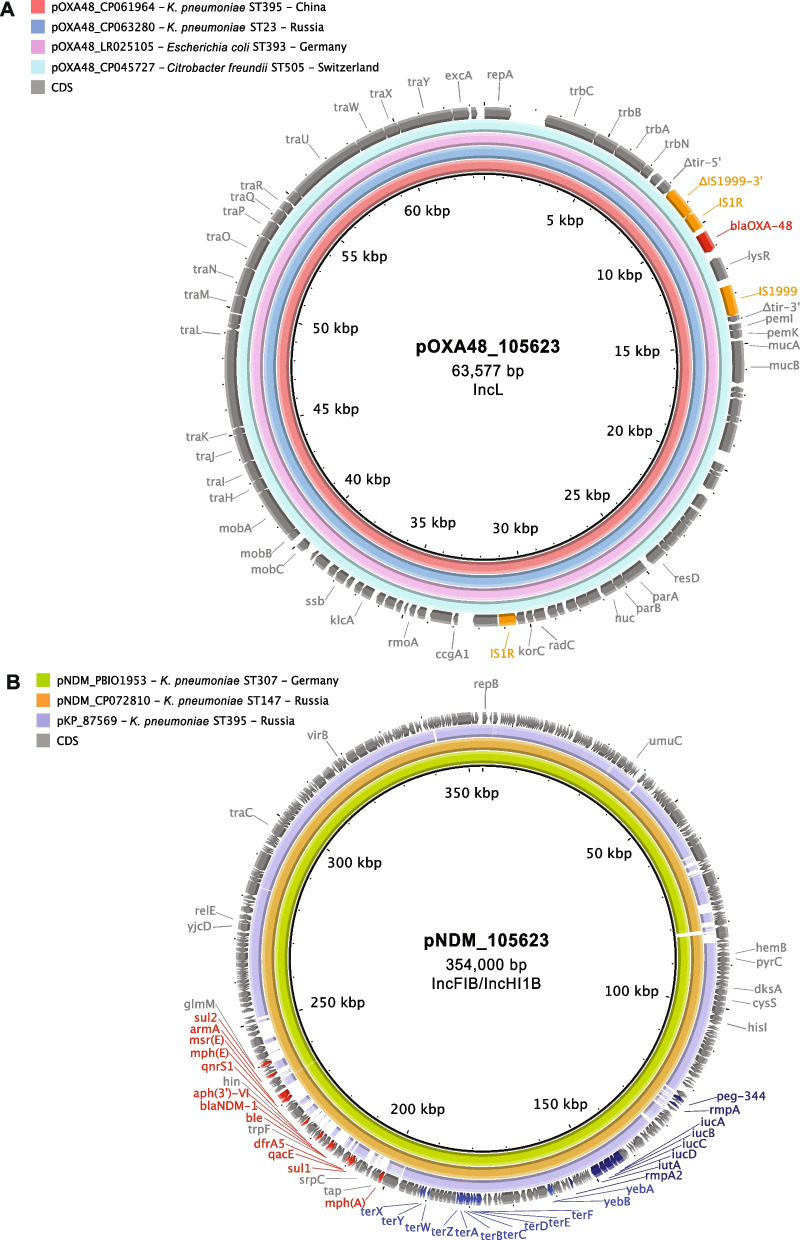
Structure of carbapenemase gene-carrying plasmids. BRIG images show similarities between plasmid sequences of selected ST395 isolates (central rings) and other published plasmid sequences identified by BLAST (concentric rings colored according to legends). Key plasmid genes are annotated by colored arrows and description: maintenance and transfer genes (gray), AMR genes (red), IS elements (orange), virulence genes (blue), and hypervirulence marker genes (deep blue). **A** IncL pOXA48-like plasmid of isolate 15623 and 87569, **B** IncFIB/IncHI1B hybrid virulence and resistance plasmids of isolates 105623 (central ring) and 87569 (outer ring)

Similarly, we determined the genetic backgrounds of aerobactin cluster genes for 114/126 isolates (90.5%). In all but two cases, we identified the presence of *iucABCD*-*iutA* on IncFIB/IncHI1B dual-replicon plasmids. Notably, 96 of 112 (85.7%) of these typical hv plasmids were found to co-harbor various antibiotic resistance determinants, including carbapenemase genes (as exemplified in Fig. 4B) and, therefore, were classified as “hybrid” or “mosaic” plasmids. A triple-replicon IncFIB/IncHI1B/IncR plasmid, which carried *iucABCD*-*iutA* and *bla*_NDM-1_ genes, was detected in one isolate, and an IncFIA/IncFII plasmid, which carried *iucABCD-iutA, rmpA*, *rmpA2*, and *peg-344*, was detected in the remaining one.

Using a combination of Illumina short-read and Oxford Nanopore long-read sequencing, we obtained the complete genomes of two Russian isolates that belonged to the largest subclade B1. One of them (87569) carried *bla*_OXA-48_ and the other (105623) had a combination of *bla*_OXA-48_ and *bla*_NDM-1_. Both isolates also possessed additional acquired resistance genes including *bla*_CTX-M-15_ and *armA*, as well as plasmid-encoded virulence factors including the aerobactin cluster (*iucABCD*-*iutA*). The *bla*_OXA-48_ gene was found as part of a Tn*1999* insertion in the chromosome of isolate 87569, and, in isolate 105623, it was located on a typical IncL pOXA48-like plasmid (Fig. 4A). BLAST analysis showed that the 63,577-bp OXA-48-encoding plasmid of 105623 (pOXA48_105623) was almost identical (99% query coverage, 99.85% identity) to the recently reported 63,589-bp plasmid of a Russian ST23 *K. pneumoniae* isolate (GenBank accession no. CP063280; [84] and was very similar to other related plasmids reported from different countries (Fig. 4). On these plasmids, the *bla*_OXA-48_ gene was carried by inverted Tn*1999.2*, which contains an insertion of IS*1R* and provides a stronger promoter for the gene expression compared to Tn*1999.1* [85, 86]. Both isolates, 87569 and 105623, also harbored large hybrid (resistance and virulence) plasmids of the IncFIB/IncHI1B type, designated pKP_87569 (307,622 bp) and pNDM_105623 (354,000 bp), respectively (Fig. 4B). These plasmids shared a common backbone/core region and important hypervirulence biomarker genes including the aerobactin cluster (*iucABCD*-*iutA*), activator of capsular polysaccharide synthesis (*rmpA2*), and tellurite resistance (*terABCDEFZ*) genes (pNDM_105623 additionally carried *rmpA* and the *peg-344* transporter genes) but differed in the content and arrangement of antibiotic resistance genes (Fig. 4B). pKP_87569 contained only four genes for chloramphenicol, aminoglycoside, and sulfonamide resistance (*catA1*, *ant(3'')-Ia*, *ant(2'')-Ia*, and *sul1*), while pNDM_105623 contained a large array of resistance genes to many antibiotic classes (*mph*(A), *msr*(E), *mph*(E), *sul1*, *sul2*, *qnrS1*, *aph(3')-VI*, *armA*, *dfrA5*) and, most notably, the *bla*_NDM-1_ carbapenemase gene. BLAST analysis revealed the highest similarity of pNDM_105623 to the previously reported plasmids of Russian ST147 *K. pneumoniae* isolate (GenBank accession no. NZ_CP072810.1; [87]) and a German ST307 *K. pneumoniae* isolate (PBIO1953; [8]) (Fig. 4B). Finally, hybrid genome assemblies of the isolates 87569 and 105623 revealed the presence of IncR plasmids that carried an ESBL gene (*bla*_CTX-M-15_) and additional resistance genes to penicillins and penicillin-inhibitor combinations (*bla*_TEM-1_ and *bla*_OXA-1_), aminoglycosides, and fluoroquinolones (*aac(6')-Ib-cr* and *qnrS1*), tetracycline (*tet*(A)), chloramphenicol (*catA1* and *catB3*), sulfonamides (*sul1*), and trimethoprim (*dfrA1*).

## Discussion

Here we describe the characteristics of *K. pneumoniae* ST395, which is an emerging high-risk clonal lineage. The investigated collection comprised 297 genomes and was geographically widely dispersed over 27 countries and four continents. More than 40% of the ST395 genomes were newly obtained as part of this study and represent Belarus, Kazakhstan, Germany, the USA, and, predominantly, Russia with the latter potentially leading to a bias in terms of interpreting geographical distributions and ST395 characteristics. Also, the fact that most studies and surveillance programs frequently use whole genome sequencing for outbreak investigations caused by MDR isolates obtained from humans might lead to underrepresentation of antibiotic-susceptible strains, which could have an additional impact on the diversity of our sample set. This limitation also applies to the interpretation of the emergence of convergent pathotypes that combine resistance with virulence. Despite these biases, our data clearly point towards high-level endemicity of *K. pneumoniae* ST395 in hospital settings across Russia and extend upon previous epidemiological reports from other groups in Russia [40, 41, 88]. In addition, our results suggest the occurrence of local outbreaks and multiple sporadic cases of ST395 infections in other countries across the world.

Expanding on the study by Wyres *et al.* [17], which revealed the descendance of ST395 lineage from ST11 (CC258) through large recombination events, we explored the role of chromosomal recombination in subsequent evolutionary processes and the intra-clade diversification of ST395. We found that the early ST395 isolates addressed by Wyres *et al.* belonged to the “original” phylogenetic clade (designated clade A in this study), which continuously spreads in multiple countries but is now being superseded by the potentially more successful clade B, at least in Europe and Asia. Overall, we assigned four distinct phylogenetic clades and eight subclades within ST395, which are well supported by hierarchical Bayesian clustering analysis of the core genome. We showed that the ST395 subclades evolved through recombination and insertion of large DNA fragments within a 1.1-Mbp chromosomal area that showed evidence of genetic exchange with unrelated clonal lineages, including *K. pneumoniae* ST377, ST147, ST437, ST15, ST896, and other yet unidentified donors. Consistent with previous reports [17, 89], our data therefore indicates that intra-clonal diversification by large-scale chromosome recombination is a common evolutionary strategy employed by typical clones of CC258 as well as ST395. Notably, the chromosomal area which showed extensive recombination in ST395 includes capsule and LPS biosynthesis loci, which likely explains the observed K and O antigen diversity between the subclades of ST395. Similar findings have been recently reported for various *Klebsiella* clonal lineages [83]. We also found that additional pan-genome diversity of ST395 is driven by the integrative conjugative element ICE*Kp*, which encodes the major virulence factor yersiniabactin [74]. The non-universal presence of ICE*Kp* and identification of different ICE*Kp* types in isolates of different ST395 subclades indicates their likely independent and frequent chromosomal acquisition and loss. Finally, we identified several prophage-related regions variably present in genomes of ST395 isolates, a trait apparently common in CC258 and other MDR clonal lineages [90].

Our results revealed endemic tendencies regarding the occurrence of certain carbapenemases. In general, the distribution of different carbapenemase genes among ST395 isolates from different countries was rather uneven and reflects the geographical endemicity patterns of certain carbapenemases. The most frequent carbapenemase genes were of the OXA-48-type and mostly distributed in Russia and Europe but also found in single isolates from the USA and China. In the retrospective surveillance of OXA-48 producing *Enterobacterales* collected between 2001 and 2011 in Europe and North Africa, *K. pneumoniae* ST395 was identified as the second most prevalent clone [91] whereas, in the very recent report from the SENTRY global surveillance, it was identified as the most prevalent genotype of OXA-48-producing *K. pneumoniae*, with the majority of isolates originating from Russia, Belarus, and Turkey [92].

Most genome sequences analyzed in this study were obtained using Illumina short-read sequencing. However, determining the plasmid content and identifying the plasmids carrying AMR and virulence genes from short-read WGS data is known to be challenging due to incomplete assembly of plasmids. The genes of interest cannot be directly assigned to a particular plasmid if found in different contigs than the plasmid marker genes. Assembly of AMR gene-carrying plasmids is especially problematic because they are usually large in size, low in copy number and frequently built as mosaic-like structures, containing mobile components that can be found in different plasmid backbones [93]. Thus, several software tools have been developed that use graph-based or reference-based approaches or their combination. Here, we used MOB-suite [80, 94] for reconstructing and characterizing plasmids carrying carbapenemase genes. MOB-suite allows “aggregation” of input sequence contigs into putative plasmids according to the reference hits obtained from the reference database of clustered complete plasmid sequences and determination of plasmid types using marker sequence databases of known replicon and relaxase genes. This software has been successfully used to characterize AMR gene-carrying plasmids in many large-scale bacterial population genetics studies, and in a recent study comparing the performance of different available plasmid prediction tools, has been shown to be the only tool to correctly reconstruct the majority of *Escherichia coli* plasmids, including large ESBL-encoding plasmids [93]. All available bioinformatics approaches, however, have important limitations and none can correctly reconstruct all plasmids from draft genome assemblies. Therefore, the data on association of carbapenemase and virulence genes with certain plasmid types presented in our study must be interpreted with caution. Also, we did not establish plasmid support for carbapenemase and aerobactin cluster genes in about 12% and 10% of *K. pneumoniae* ST395 genome sequences.

Consistent with many previous reports, in our study, *bla*_OXA-48-like_ genes were predominantly found on IncL and IncM2 plasmids, which are known as primary vehicles for *bla*_OXA-48_ dissemination worldwide [95]. *bla*_NDM_ genes were often harbored by multi-replicon IncFIB/IncFII plasmids and hybrid (virulence and resistance) IncFIB/IncHI1B plasmids [87, 96], and *bla*_KPC-3_ genes were harbored by pKPC_Kp02-like plasmids, which has also been previously described [97].

To further characterize the typical carbapenemase-encoding plasmids, we obtained the complete genome sequences of two Russian isolates from the largest subclade B1. The OXA-48-encoding plasmid of one of those isolates was almost identical to the IncL plasmids reported previously in different strains of *Enterobacterales*: *K. pneumoniae* ST23 from Russia, *K. pneumoniae* ST395 from China, *E. coli* ST393 from Germany and *Citrobacter freundii* ST505 from Switzerland (Fig. 4A). Literature suggests that the Chinese isolate originated from a human and was collected in 2015, whereas the Swiss one was obtained from the grass surroundings of a veterinary clinic in 2019. For the German isolate, neither the collection date nor the origin location was available. However, the German sequence has been previously described in several publications [84, 98, 99]. In a recent report on a *K. pneumoniae* ST23 outbreak in Göttingen, Germany, in 2013, it was shown, that at least one outbreak isolate seemingly carried an identical plasmid [98]. Note that the occurrence of many carbapenemase-negative genomes in multiple countries likely indicates that production of these enzymes is not the only driver of successful dissemination of ST395, however. ESBL genes, mostly of CTX-M-type, were frequently present (87%) and other types occurred less often (< 3%). The simultaneous occurrence of ESBL and carbapenemase genes is not unusual and consistent with previous findings. In addition to these, partly last-resort drug resistances, the presence of 16S rRNA methyltransferases intensifies the overall tense situation, where life-threatening infections with MDR *Klebsiella* cannot be reliably treated [16]. Therefore, the presence of 16S rRNA methyltransferase genes in > 38% of the investigated isolates is alarming, especially in Russia, where ~ 40% of the isolates were positive for 16S rRNA methyltransferase genes. These rates are high compared to revealed rates from Spain (5%) and the UK (∼16–18%), and similar to those in Asian countries (50%) [16]. We only found one genome with mobile colistin resistance (*mcr*) gene, which seemingly mirrors the current global situation of mostly colistin-susceptible ST395 isolates. However, we cannot exclude phenotypic colistin resistance conveyed through chromosomal adaptations. Given that colistin is still frequently used in veterinary medicine, it is not surprising that the *mcr-*carrying isolate in our study was obtained from a chicken.

ST395 was initially reported as non-hypervirulent type associated with multiple antibiotic resistances in nosocomial isolates. However, considering recent findings [41, 88, 100], we explored the acquired hv markers and their plasmid backbones. In total, we found 130 (43.8%) isolates carrying at least one hv marker gene. We found 96 isolates with plasmids classified as “hybrid” or “mosaic” plasmids, co-harboring hv and various AMR genes. As our sample set is biased towards MDR *K. pneumoniae*, however, these convergent genotypes might be overrepresented in this study, possibly not accurately reflecting the prevalence in larger datasets that contain a higher number of susceptible isolates.

We also confirmed that, in ST395, aerobactin was more common than other hv biomarkers, as it has been shown for other *K. pneumoniae* clonal lineages [101]. Interestingly, most of the isolates co-harboring virulence and resistance genes belonged to clade B, mostly of capsular type K2. This adds to the results of Lazareva from 2020 [41]. The convergence of ST395 hypervirulence and MDR has already been highlighted by some reports, especially in recent years [41, 88, 100]. Finding a similar hybrid (resistance and virulence) plasmid in three Russian isolates (two ST395 and one ST147) and one German (ST307) isolate indicates transmission of AMR-encoding plasmids among ST395 and other *Klebsiella* sequence types.

## Conclusions

This study revealed the ability of ST395 to evolve and diversify into a highly successful, antibiotic-resistant, and virulent clonal lineage through both chromosomal recombination and acquisition of multiple extrachromosomal elements and provided evidence for its dissemination across countries and continents. Our results also suggest that controlling the further spread of this and other pathogenic bacterial lineages will remain challenging.

## Supplementary Information

**Additional file 1:** **Table S1.** Characteristics of the isolates from the ST395 dataset. **Table S2.** Comparison of cgMLST profiles of isolates representing ST395 subclades. **Table S3.** Location of carbapenemase genes.

**Additional file 2.** Calculation of mean recombination counts. Formula for calculation of mean recombination counts.

**Additional file 3.** Detailed Methods. Overview of used bioinformatics tools and parameters.

**Additional file 4: **Identification of large recombination events and putative donors. **Table S4.** Large (>100 kbp) chromosome recombination events predicted by Gubbins and the putative donors identified by core-SNP phylogenetic analysis from the expanded genome dataset. **Fig. S1.** An example tree (A) and subtree (B) used to identify the ST437 as a putative donor for recombination event 13 in ST395 subclade D2.

## Data Availability

All raw and assembled Illumina and Oxford Nanopore sequence data are available from the European Nucleotide Archive under the study accession nos. PRJEB49683 (https://www.ebi.ac.uk/ena/browser/view/PRJEB49683) [46] and PRJEB53512 (https://www.ebi.ac.uk/ena/browser/view/PRJEB53512) [49]. Assemblies of the US samples have been deposited with links to BioProject accession number PRJNA849970 [50] in the NCBI BioProject database (https://www.ncbi.nlm.nih.gov/bioproject/PRJNA849970). Individual accession numbers for raw reads and/or de novo assemblies are also available in the Additional file 1: Table S1. The entire ST395 genome dataset can be accessed as a Microreact project at https://microreact.org/project/uyaJweHqXXxdVudHU3QDwW.

## Abbreviations

AMR: Antimicrobial resistance
CC: Clonal complex
ESBL: Extended-spectrum beta-lactamase
hvKp: Hypervirulent *Klebsiella pneumoniae*
hv: Hypervirulent
IAC: Institute of Antimicrobial Chemotherapy
MDR: Multidrug-resistant/multidrug resistance
MFS: Major facilitator superfamily
MLST: Multilocus sequence typing
ORF: Open reading frame
RND: Resistance-nodulation-cell division
ST: Sequence type
