# Supplementary material for: Genomic analysis of the international high-risk clonal lineage Klebsiella pneumoniae sequence type 395
Source: Genome Med. 2023 Feb 13;15:9. doi: 10.1186/s13073-023-01159-6 (PMC9926764; doi:10.1186/s13073-023-01159-6)
Supplement: Supplementary file 2 — Additional file 2. Calculation of mean recombination counts. Formula for calculation of mean recombination counts. [file 13073_2023_1159_MOESM2_ESM.docx]

Additional file 2

**Genomic analysis of the international high-risk clonal lineage *Klebsiella pneumoniae* sequence type 395**

Shaidullina ER^*^, Schwabe M^*^ *et al.*

^*^shared first authorship

**Calculation of mean recombination counts**

Consider the set

$$X=\left\{ \left( r, p, v \right) | r\in\left\{ 1,\cdots,e \right\}, p\in\left\{ 1,\cdots, l \right\}, v\in\left\{ 0,1 \right\} \right\},$$

where $r$ – recombination event index, $p$ – position in the chromosome, $v$ – categorical value, it is equal to $0$, if the position was not within the recombination event and it is equal to $1$, if it was, $e$ – total number of recombination events that was extracted from the Gubbins output file, $l$ – length of the chromosome.

Example 1:

$$\left( 1, 1, 1 \right)$$

$$\left( 2, 1, 1 \right)$$

$$\vdots$$

$$\left( 26, 1000, 1 \right)$$

$$\left( 27, 1000, 1 \right)$$

$$\left( 28, 1000, 1 \right)$$

$$\left( 29, 1000, 1 \right)$$

$$\left( 30, 1000, 1 \right)$$

Let us define

$$w_{i}=\left| \left\{ \left( r, p, v \right)\in X|p=i, v=1 \right\} \right|,$$

so $w_{i}$ is a sum of all $1$’s in the position $i$ through all the recombination events.

Example 2:

$$w_{1}=2$$

$$w_{2}=0$$

$$\vdots$$

$$w_{1000}=5$$

Hence the mean recombination counts per $n$ positions could be calculated as

$$\bar{w_{j}}=\frac{w_{1+\left( j-1 \right)\times n}+w_{2+\left( j-1 \right)\times n}+\cdots+w_{n+(j-1)\times n}}{n},$$

where $j\in\left\{ 1, 2, \cdots, \frac{l}{n} \right\}, n$ was chosen to be equal to 1000.

Example 3:

$$\frac{(2+0+\cdots+5)}{1000}$$
