# Supplementary material for: Genomic analysis of the international high-risk clonal lineage Klebsiella pneumoniae sequence type 395
Source: Genome Med. 2023 Feb 13;15:9. doi: 10.1186/s13073-023-01159-6 (PMC9926764; doi:10.1186/s13073-023-01159-6)
Supplement: Supplementary file 3 — Additional file 3. Detailed Methods. Overview of used bioinformatics tools and parameters. [file 13073_2023_1159_MOESM3_ESM.docx]

Additional file 3

**Genomic analysis of the international high-risk clonal lineage *Klebsiella pneumoniae* sequence type 395**

Shaidullina ER^*^, Schwabe M^*^ et al.

^*^shared first authorship

**Detailed Methods: Overview of used bioinformatics tools and parameters**

**MLST**

mlst --threads 8 <in.fasta>

**Trimmomatic**

Trimmomatic PE -threads 8 -phred 33 read1 read2 -baseout outfile -trimlog logfile ILLUMINACLIP:<adapterfile.fasta>:2:30:10 LEADING:3 TRAILING:3 SLIDINGWINDOW:4:15 MINLEN:36

Adapters included:

Illumina Paired End Adapter 1/2

Illumina Paired End PCR Adapter 1/2

Illumina Paired End Sequencing Primer 1/2

Illumina NlaIII expression Adapter 1/2

Illumina NlaIII expression PCR Primer 1/2

Illumina NlaIII expression Sequencing Primer

Illumina Multiplexing Adapter 1/2

Illumina Multiplexing PCR Primer 1.01

Illumina Multiplexing PCR Primer 2.01

Illumina Multiplexing Read1/2 Sequencing Primer

Illumina Multiplexing Index Sequencing Primer

TruSeq Universal Adapter

TruSeq Adapter, Index 1-12

**Kraken**

kraken2 --memory-mapping --db KRAKEN_DB --gzip-compressed --paired reads1 reads2 --output read.RESULTS_P.txt --report read.REPORT.txt --threads 8

**Krankentoolkit**

extract_kraken_reads.py -k read.RESULTS_P.txt -s1 reads1 -s2 reads2 -o reads1 -o2 reads2 -r report read.REPORT.txt --include-children --fastq-output --exclude -t 2759

**SPADES**

spades.py -1 reads1 -2 reads2 --cov-cutoff off --tmp-dir tmp --careful -o outdir -t 8

**Guppy**

guppy_basecaller -i fast5_dir -s fastq_dir --config sup.cfg --flowcell FLO-MIN106 --kit SQK-LSK109 --barcode_kits EXP-NBD104 --trim_barcodes --compress_fastq --detect_mid_strand_barcodes --min_score_mid_barcodes 60 -x auto

**Trycycler**

filtlong --min_length 1000 --keep_percent 95 input_reads.fastq.gz > reads.fastq

trycycler subsample --reads reads.fastq --out_dir read_subsets --genome_size 5.5m

flye --nano-hq reads.fastq --threads 90 --out-dir assembly

miniasm_and_minipolish.sh reads.fastq 90 > assembly.gfa

any2fasta assembly.gfa > assembly.fasta

raven --threads 90 reads.fastq > assembly.fasta

trycycler cluster --assemblies assemblies/*.fasta --reads reads.fastq --out_dir trycycler

trycycler msa --cluster_dir trycycler/cluster_00*

trycycler partition --reads reads.fastq --cluster_dirs trycycler/cluster_*

trycycler consensus --cluster_dir trycycler/cluster_00*

**Medaka**

medaka_consensus -i reads.fastq -d 7_final_consensus.fasta -o medaka -m r941_min_sup_g507 -t 90

**Polypolish**

polypolish consensus.fasta alignments_1.sam alignments_2.sam > polypolish.fasta

**POLCA**

polca.sh -a polypolish.fasta -r "1.fastq.gz 2.fastq.gz" -t 16 -m 1G

**Parsnp**

parsnp -r ref.fasta -d dir_genomes -p 30 -c -x -o output_dir -v

**BAPS/fastBAPS**

Function optimise_prior with parameter type = "optimise.symmetric"

**Snippy/Gubbins/FastTree**

snippy --cpus 86 --outdir dir --ref ref.fasta --R1 reads1 --R2 reads2 --report

snippy-core --ref ref.fasta output_dir

gubbins.py aln.file --prefix data --threads 94 --verbose

FastTree -boot 100 input_file > output_file

**Prokka**

prokka genome.fasta --outdir dir --genus Klebsiella --usegenus --cpus 88

**AMRFinderPlus**

amrfinder -p prokka_proteins_output.faa -g prokka_annotation.gff -n genome.fasta -O Klebsiella  --plus

**Abricate**

abricate --minid 90 --mincov 60

**Kleborate**

kleborate --all -o kleborate -a genome.fasta

**ISFinder online**

Default

Blastn, wordsize=11, evalue=10, gapOpen = Existence:5 Extension:2

**Phaster online**

default

**MOB Suite**

mob_recon --infile assembly.fasta --outdir my_out_dir
