# Supplementary material for: Genomic analysis of the international high-risk clonal lineage Klebsiella pneumoniae sequence type 395
Source: Genome Med. 2023 Feb 13;15:9. doi: 10.1186/s13073-023-01159-6 (PMC9926764; doi:10.1186/s13073-023-01159-6)
Supplement: Supplementary file 4 — Additional file 4: Identification of large recombination events and putative donors. Table S4. Large (>100 kbp) chromosome recombination events predicted by Gubbins and the putative donors identified by core-SNP phylogenetic analysis from the expanded genome dataset. Fig. S1. An example tree (A) and subtree (B) used to identify the ST437 as a putative donor for recombination event 13 in ST395 subclade D2. [file 13073_2023_1159_MOESM4_ESM.docx]

Additional file 4

**Genomic analysis of the international high-risk clonal lineage *Klebsiella pneumoniae* sequence type 395**

Shaidullina ER^*^, Schwabe M^*^ *et al.*

^*^shared first authorship

**Identification of large recombination events and putative donors**

The complete genome of the PBIO1951 isolate was used as the reference genome. All other ST395 genomes from the dataset were mapped to the reference genome using Snippy. The putative regions of recombination were predicted by Gubbins. The Gubbins output files were used to calculate the mean recombination count per base, and to extract and annotate large (>100 kbp) recombination regions in the chromosome. The method described by Comandatore *et al.* (Genome Biol Evol. 2019;11(11):3240-3251) was then used to identify the putative donors of the large recombination events detected within ST395. Briefly, a total of 1,303 complete genome assemblies of *K. pneumoniae* isolates representing different STs were retrieved from the Patric database (<https://www.patricbrc.org>). They were merged to the ST395 dataset and aligned to the reference genome of PBIO1951. The core SNPs were called again using Snippy and those corresponding to each recombination region of >100 kbp predicted by Gubbins were extracted and subjected to separate phylogenetic analyses using the FastTree tool (<http://www.microbesonline.org/fasttree/>) with 100 resamples. Each resulting tree was manually analyzed as follows: i) the ST395 recipients of the recombination were identified on the tree and ii) when a monophylum including all the recipients and one or more non-ST395 strains was detected, the latter were considered as putative donors of the recombination (see below: Table S4, Figure S1).

**Table S4.** Large (>100 kbp) chromosome recombination events predicted by Gubbins and the putative donors identified by core-SNP phylogenetic analysis from the expanded genome dataset.

| **Recomb. event** | **Genome position, start – end** | **Length [bp]** | **Neg log likelihood** | **SNP count** | **ST395 subclade** | **Putative donor** |
| --- | --- | --- | --- | --- | --- | --- |
| 68 | 1,092,537 – 1,259,900 | 167,363 | 17,373.31 | 1206 | C | ST147 (CG147) |
| 70 | 1,265,104 – 1,460,425 | 195,321 | 9,935.42 | 1005 | C | ST147 (CG147) |
| 69 | 1,575,484 – 1,753,682 | 178,198 | 13,579.09 | 1135 | C | ST147 (CG147) |
| 432 | 1,610,810 – 1,864,778 | 253,968 | 10,750.43 | 2334 | D3 | ST15 |
| 13 | 1,652,788 – 2,041,774 | 388,986 | 9,548.28 | 1473 | D2 | ST437 |
| 19 | 1,729,056 – 1,979,109 | 250,053 | 1,661.32 | 478 | A2 | ST377 |
| 433 | 1,901,126 – 2,194,712 | 293,586 | 3,658.95 | 1199 | D3 | ST11 |
| 24 | 1,936,995 – 2,038,031 | 101,036 | 2,271.78 | 415 | D1 | ST896 |
| 14 | 2,048,115 – 2,236,105 | 187,990 | 4,784.78 | 812 | D2 | ST11 |

**A**


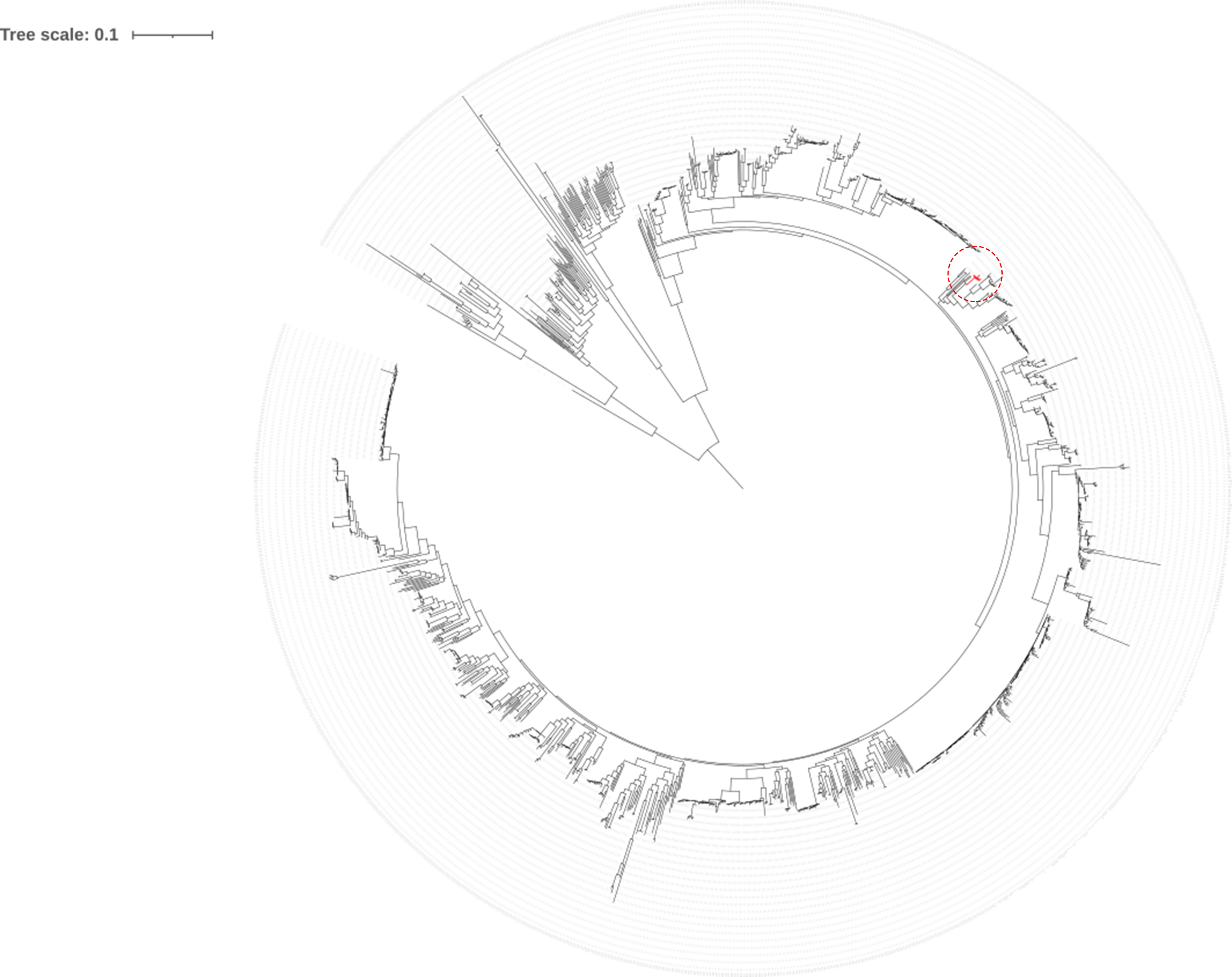


**B**


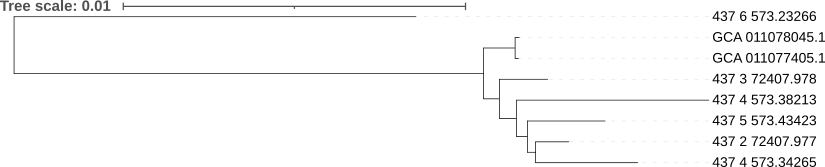


**Figure S1.** An example tree (A) and subtree (B) used to identify the ST437 as a putative donor for recombination event 13 in ST395 subclade D2.
